# Immigrant ancestry and birthweight across two generations born in Sweden: an intergenerational cohort study

**DOI:** 10.1136/bmjgh-2021-007341

**Published:** 2022-04-10

**Authors:** Siddartha Aradhya, Srinivasa Vittal Katikireddi, Sol P Juárez

**Affiliations:** 1 Demography Unit (SUDA) and Department of Sociology, Stockholm University, Stockholm, Sweden; 2 Centre for Economic Demography (CED), Lund University, Lund, Sweden; 3 MRC/CSO Social and Public Health Sciences Unit, University of Glasgow, Glasgow, UK; 4 Department of Public Health Sciences, Stockholm University, Stockholm, Sweden; 5 Centre for Health Equity Studies (CHESS), Stockholm University/Karolinska Institutet, Stockholm, Sweden

**Keywords:** epidemiology, cohort study, public health, nutrition

## Abstract

**Introduction:**

Differences in birthweight are often seen between migrants and natives. However, whether migrant-native birthweight inequalities widen, narrow or remain persistent across generations when comparing the descendants of immigrants and natives remains understudied. We examined inequalities in birthweight of mothers (G2) and daughters (G3) of foreign-born grandmothers (G1) compared with those of Swedish-born grandmothers.

**Methods:**

We used population registers with multigenerational linkages to identify 314 415 daughters born in Sweden during the period 1989–2012 (G3), linked to 246 642 mothers (G2) born in Sweden during 1973–1996, and to their grandmothers (G1) who were Swedish or foreign-born. We classified migrants into non-western, Eastern European, the rest of Nordic and Western. We used multivariable methods to examine mean birthweight and low birthweight (<2500 g; LBW).

**Results:**

Birthweight between individuals with Swedish background (G1) and non-western groups increased from -80 g to -147 g between G2 (mothers) and G3 (daughters), respectively. Furthermore, the odds of LBW increased among the G3 non-western immigrants compared with those with Swedish grandmothers (OR: 1.38, 95% CI 1.12 to 1.69). Birthweight increased in both descendants of Swedes and non-western immigrants, but less so in the latter (83 g vs 16 g).

**Conclusion:**

We observed an increase in birthweight inequalities across generations between descendants of non-western immigrants and descendants of Swedes. This finding is puzzling considering Sweden has been lauded for its humanitarian approach to migration, for being one of the most egalitarian countries in the world and providing universal access to healthcare and education.

**Key questions**

**What is already known on this topic**

- There are documented differences in birthweight among the descendants of white and non-white immigrants in several contexts, but little is known about how these inequalities developed over generations.
- Studies comparing birthweight outcomes between first-generation and second-generation immigrants showed little change in birthweight within immigrant groups, but they did not assess inequalities relative to the native populations.

**What this study adds**

- Birthweight markedly increased between mothers and daughters born in Sweden for all origin groups except for the descendants of non-western immigrants.
- The third-generation descendants of non-western immigrants experience substantial birthweight inequalities compared with their Swedish native counterparts.

**How this study might affect research, practice or policy**

- Our study suggests that countries where large-scale migration is a recent phenomenon are at risk of developing similar racial inequalities in birthweight as seen in countries with long migration histories, such as the UK, USA and Brazil.

Although large and persistent ethnic inequalities in perinatal health are well documented worldwide, inequalities among immigrants show a less clear pattern.^1^ Studies on birthweight among foreign-born individuals show both advantages^2–4^ and disadvantages^5^ compared with the host native population, although it remains unclear the extent to which such variation is explained by migrants’ health or by varying health standards of the population in the receiving countries.^5 6^ Though less investigated, studies comparing birthweight by mother’s duration of residence in the host country show that immigrants have higher risk of low birthweight with no improvement^7^ or an increased risk with longer residence (over 10 years).^8 9^ This suggests that increased exposure to the host society may have deleterious effects on the health of immigrants that can be transmitted over generations. An intergenerational perspective is needed to understand how health inequalities develop in society as well as to link research on ethnic and immigrant inequalities in perinatal health.

While efforts to compare birthweight outcomes between the first-generation and second-generation immigrants exist, there remain important methodological and analytical limitations. A previous systematic review^10^ found negligible birthweight differences over generations within origin groups. However, to date, no studies compare intergenerational birthweight changes to the majority host population, thus lacking an evaluation of inequalities. In addition, studies are restricted to the USA and the UK with limited generalisability.

Sweden is a unique context to study the development of inequalities among migrants due to the large and diverse immigrant population. Moreover, the country’s humanitarian approach to immigration, generous welfare state and equal access to healthcare among residents offer an opportunity to examine how favourable public health system can equalise health in an increasingly diverse population. To date, studies show that immigrants from less developed origins display approximately −100 g differences relative to Swedes, with no change by duration of residence.^7^ However, no studies have examined whether birthweight among the second-generation immigrants improves, remains constant or declines relative to their native counterparts in Sweden.

Using total population register data with multigenerational linkages, we examined birthweight differences between Swedish-born mothers (generation 2, G2) and daughters (G3) of migrant grandmothers compared with those with Swedish grandmothers (G1). We use the descendants of Swedish grandmothers as the reference group in order to assess inequalities over generations and compare different regions of origin.

## Methods

### Data

The data are obtained from the Swedish Interdisciplinary Panel (SIP) administered by the Centre for Economic Demography at Lund University. The SIP was constructed by linking information between various administrative registers, including the Medical Birth Register (MBR), which started in 1973,^11^ the Total Population Register, Income and Taxation Register, the Educational Register and the Multi-Generation Register (MGR)^12^ through unique personal identification numbers.^13^

### Study population

**Figure 1:**
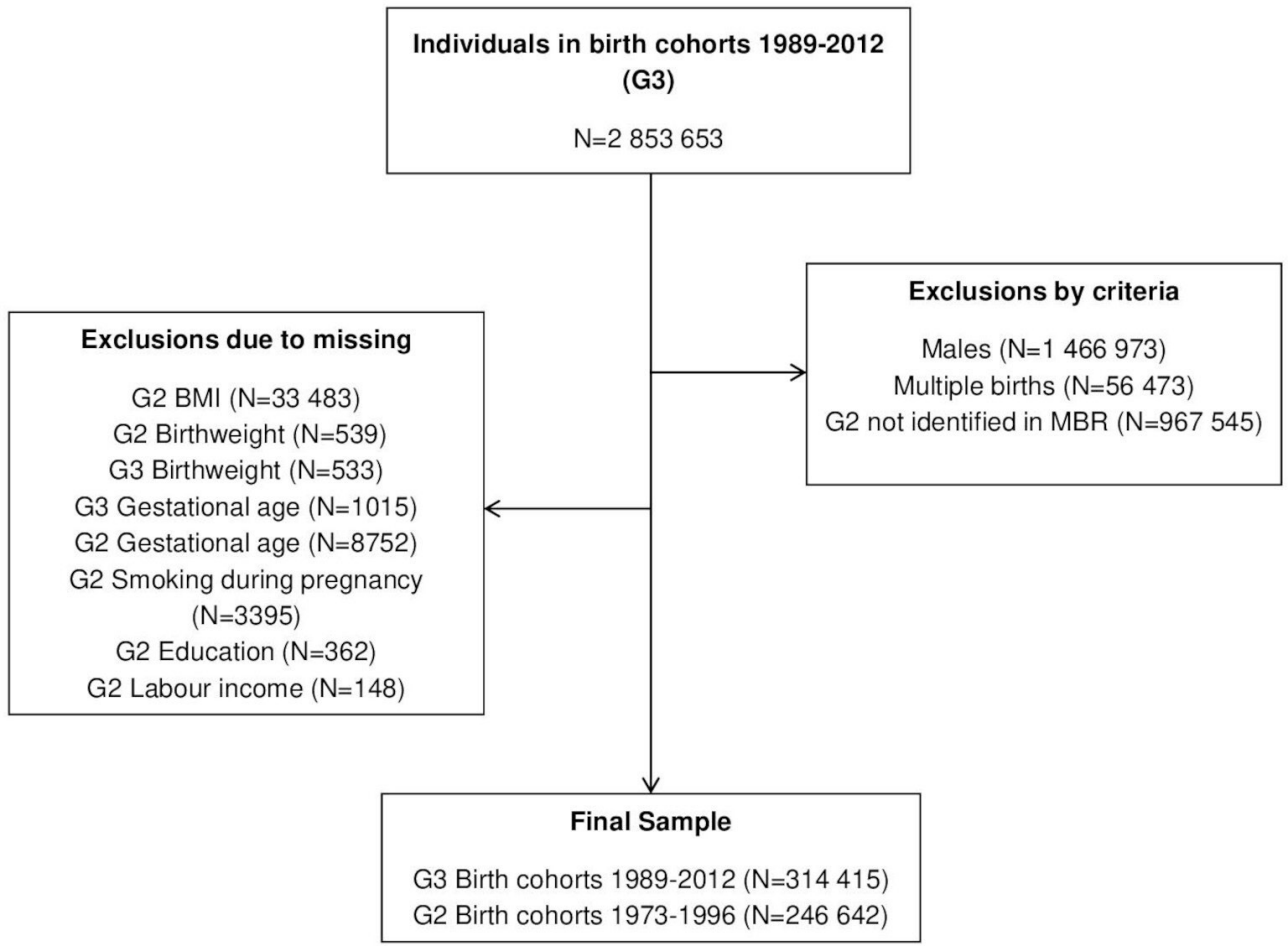
Selection flow and study population. MBR, Medical Birth Register.

Through the MBR, girls born in Sweden between 1989 and 2012 (the index population, G3) were linked to their mothers (G2) born in Sweden between 1973 and 1996 and through the MGR, their maternal grandmothers, who were either native-born or foreign-born (G1). The exclusion of G3 women because G2 was unidentified in the MBR occurred because either (1) G2 was born before 1973 or (2) G2 was not born in Sweden. As a result, full matrilineal linkages were identified for G3 individuals whose G2 mothers were 39 years of age or younger and born in Sweden. We excluded the 1989 and 1990 birth cohorts from the analysis due to missing information on mothers’ early pregnancy weight and height in the MBR. Similarly, we excluded observations due to missing information in all of the covariates. We observed no evidence of a systematic differential pattern of missing data by maternal grandmothers’ origins, which was expected as the study population was born in Sweden. The study population included 246 642 G2 mothers and 314 415 live singleton G3 daughters. See figure 1 for the selection flow.

### Outcome variable and covariates

The outcomes were birthweight in grams and low birthweight (<2500 g) drawn from the MBR, which contains ~99% of all births that occur in Sweden.^14^ The exposure was grandmothers’ (G1) country or region of birth. We categorised the G1 country/region of birth into five main groups: Sweden, non-Western countries (ie, Chile, Turkey, Lebanon, Iran and Iraq or East Africa, the rest of Africa, South America (excluding Chile) and Asia); Eastern European countries (ie, Poland, the former Soviet Union, former Yugoslavia and other European countries not in the EU-27); western countries (ie, the EU-27, the USA and Oceania); and finally, the rest of the Nordic countries (ie, Finland, Norway and Denmark). The non-western and Eastern European groups are largely comprised of migrants from countries in conflict, namely, asylum seekers and refugees, while western countries are represented by labour migrants. Migration to Sweden after the early 1970s has been comprised predominantly of refugees, asylum seekers and family reunification immigrants.^15 16^ Based on historical roots, the rest of Nordic countries are considered in a separate category.

G2 covariates were as follows: birthweight (grams); height (centimetres); early pregnancy Body Mass Index, BMI (linear); age at birth (<20, 20–24, 25–34, ≥35); gestational age (<37 weeks, 37–42 weeks, >42 weeks); smoking during pregnancy (non-smoker, 1–9 cigarettes/day, ≥10 cigarettes/day); labour income (quintiles) and education (less than tertiary, tertiary or higher). G3 covariates included birth order (1, 2, 3+); gestational age (<37 weeks, 37–42 weeks, >42 weeks) and year of birth (linear).

### Statistical analysis

We performed two sets of analyses. First, we fitted linear regression models to estimate beta coefficients (β) and their 95% CIs in order to measure average birthweight differences for G2 and G3, independently, by G1 region of birth relative to Swedish natives. Predicted values were presented to compare average birthweight differences between G3 and G2 within each origin category. We also fitted logistic models to estimate ORs and 95% CI for low birthweight (<2500 g). All models were estimated with robust SEs to account for the presence of multiple daughters born to the same mother.

Second, we compared G3 birthweight by G1 origin relative to the native Swedish population to assess the intergenerational transmission of birthweight inequalities. We fitted random effect linear regression models to account for the hierarchical structure of the data (daughters G3 nested within mothers G2).

Four model specifications were estimated for birthweight in grams. Model 1 adjusted for gestational age estimates the overall birthweight differences by grandmother’s (G1) region of birth, adjusting for the portion of the differences that are due to gestational age. Model 2 is an extension of model 1, including year of birth and birth order. Model 3 additionally includes G2 birthweight, gestational age, height and BMI. The inclusion of BMI and height in the same model allowed for the control of body composition.^17^ Models 2–3 were fitted to assess the role of compositional factors in explaining birthweight differences between groups. Model 4 additionally adjusted for socioeconomic characteristics (education and income), age at childbirth and smoking during pregnancy—as the latter two are not equally socially patterned across groups. Model 4 was fitted to examine potential mediation. Additionally, we fitted two models for low birthweight. Model 1 adjusted for gestational age and model 2 corresponding to model 4 above.

Two sets of sensitivity analyses were conducted. First, we excluded preterm births (<37 gestational weeks) to assess whether our findings were impacted by changes in preterm deliveries between generations, possibly due to medical improvements that increased the viability of small babies. Second, we replicated the analyses using the least aggregated country or region of birth groupings provided by Statistics Sweden to assess the validity of our classifications.

### Patient and public involvement

Neither patients nor the public were directly involved in this review.

## Results

Table 1 describes the study population by G1 region of birth and the covariates in each generation. Roughly 9% of the population is of non-Swedish ancestry. The largest non-Swedish group was the rest of the Nordic, followed by non-western countries. While small group differences in BMI and height were found among G2, substantial differences in age at childbirth were observed with the non-western group being younger relative to other populations. The rest of Nordic group showed the largest proportion of mothers who smoked during pregnancy (approximately 15%), whereas the non-western group had the lowest levels in socioeconomic measures (tertiary education and labour income). Among G3 births, there was an even distribution of birth order across groups. Mean birthweight increased among all groups from G2 to G3, although to varying degrees (21 g and 87 g increase from G2 to G3 among non-western and Rest of Nordic, respectively), and low birthweight (LBW) decreased.

**Table 1:** Characteristics of the study population by grandmothers’ (G1) region/country of birth

G1: Grandmothers' region/country of origin		Non-Western	Eastern European	Western	Rest of Nordic	Sweden
		%/mean (SD)	%/mean (SD)	%/mean (SD)	%/mean (SD)	%/mean (SD)
G2: Mothers (born 1973–1996) N=246 642						
Post-secondary education	No	66.16	58.69	47.69	59.72	50.17
	Yes	33.84	41.31	52.31	40.28	49.83
Gestational age	<37 weeks	5.34	5.56	4.69	4.48	3.79
	37–42 weeks	93.07	92.10	92.67	92.53	93.00
	>42 weeks	1.59	2.34	2.65	2.99	3.18
Height		164 (6)	167 (6)	166 (6)	166 (6)	167 (6)
LBW		4.22	4.71	4.38	4.13	3.79
Birthweight (grams)		3336 (492)	3359 (528)	3368 (511)	3417 (529)	3434 (521)
**N**		3463	3292	2646	12 570	224 671
G3: Daughters (born 1989–2012) N=314 415						
Birth order	1	57.27	56.46	54.93	52.67	54.04
	2	31.47	33.55	35.31	34.40	35.23
	3+	11.26	9.99	9.76	12.94	10.73
Maternal age*	Less than 20	5.39	3.36	2.36	3.71	2.23
	20–24	29.64	19.18	17.02	23.01	18.71
	25–29	39.31	37.50	34.55	36.52	37.42
	30–34	22.24	32.14	35.88	29.33	33.48
	35+	3.42	7.82	10.19	7.42	8.15
Maternal smoking during pregnancy*	Non-smoker	88.67	86.26	89.24	84.71	90.80
	1–9 cigarettes/day	9.36	10.84	7.92	10.97	7.02
	>10 cigarettes/day	1.97	2.90	2.84	4.32	2.18
Maternal income (quintiles)*	1 Top	8.58	13.47	15.42	9.72	12.76
	2	22.24	30.36	31.26	29.24	33.60
	3	42.77	38.21	38.66	44.42	41.94
	4	18.13	11.23	10.13	11.92	9.03
	5 Bottom	8.28	6.73	4.53	4.71	2.67
BMI*		24 (5)	24(4)	24 (4)	25 (5)	24 (5)
Gestational age	<37 weeks	4.30	5.07	4.32	5.12	5.32
	37–42 weeks	95.65	94.79	95.65	94.79	94.61
	>42 weeks	0.05	0.15	0.03	0.09	0.07
LBW		3.58	3.36	3.05	3.13	3.32
Birthweight (grams)		3357 (499)	3427 (506)	3442 (506)	3504 (542)	3494 (537)
**N**		4325	4104	3308	16 179	286 499

*Corresponds to maternal information that varies between births.

LBW, low birthweight.

**Figure 2:**
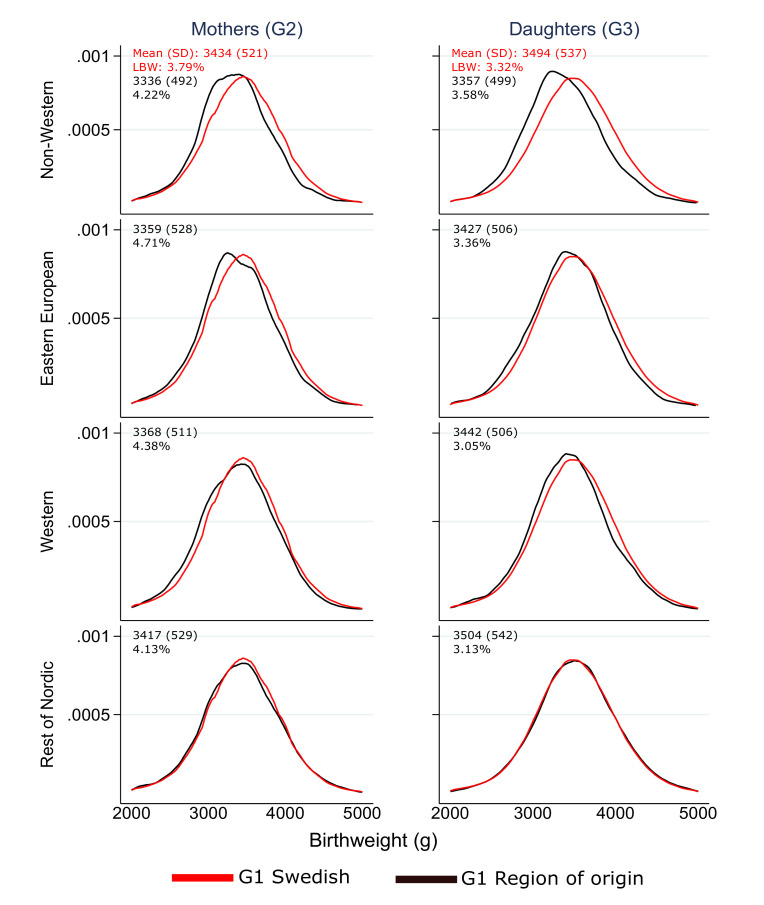
Birthweight distributions (Kernel density plots) for mothers (G2) and daughters (G3) born in Sweden by grandmothers’ (G1) region of origin relative to the Swedish Natives. LBW, low birthweight.

Figure 2 displays the birthweight distribution for mothers (G2) and daughters (G3) by grandmother’s (G1) region of origin relative to the corresponding population with Swedish-born G1. Among the G2 population, the native Swedish origin group had the highest mean birthweight (3434 g), with the non-western group displaying the lowest mean birthweight (3336 g). In G3, only the rest of the Nordic group displayed a higher mean birthweight than the Swedish-origin group (3504 g vs 3494 g). All groups experienced a uniform increase in birthweight distributions (mean and SD), but this increase was modest for the non-western group, which leads to an overall divergence from the native population. Mean birthweight adjusted for gestational age (predicted values from model 1) show that all groups have experienced an increase between G2 and G3. The increase was largest among the Nordics (102 g), followed by native Swedes (83 g), Western (76 g), Eastern Europeans (69 g) and non-western (16 g).

**Figure 3:**
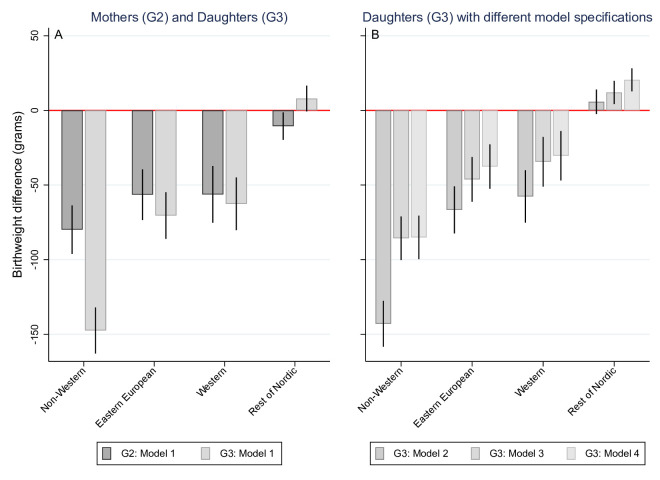
(A) Birthweight differences for mothers (G2) and daughters (G3) by grandmothers’ (G1) regions of origin relative to native Swedes G1. (B) Birthweight differences for daugthers (G3) by grandmothers’ (G1) regions of origin relative to native Swedes G1 with different model specifications. Beta coeficients with 95% CIs from linear regression models.

Figure 3 (estimates presented in online supplemental table 1) shows birthweight differences by grandmother’s (G1) region of birth among G2 and G3 adjusted for gestational age, respectively. All G2 groups had lower average birthweight than natives and, with the exception of those from the rest of the Nordics, also in G3. When comparing groups across generations to natives, two patterns emerged. On the one hand, there was a divergence in birthweight among the non-western group across generations, changing from -80 g difference in G2 (95% CI −96 g to −64 g) to -147 g difference in G3 (95% CI −163 g to −132 g) relative to natives. On the other hand, individuals with rest of Nordic ancestry increased from −11 g (95% CI −19 g to −1 g) in G2 to an 8 g difference in birthweight (95% CI −1 g to 17 g) in G3 relative to natives. The Eastern European and Western groups exhibited a moderately divergent trend from native birthweight levels, although with overlapping CIs (for Eastern Europeans ancestry, from β_G2_ −56 g 95% CI −73 g to −39 g to β_G3_ −70 g 95% CI −86 g to −55 g; and for western ancestry, from β_G2_ −56 g 95% CI −75 g to −37 g to β_G3_ −63 g 95% CI −80 g to −45 g). The non-western group relative to natives also displayed increased odds of low birthweight in G3 (OR 1.38 95% CI 1.12 to 1.69) (table 2). Sensitivity analysis restricted to term births in G2 and G3 showed consistent results for both mean and low birthweight (online supplemental tables 2 and 3).

10.1136/bmjgh-2021-007341.supp1 Supplementary data

**Table 2:** Low birthweight (<2500 grams) differences among mothers (G2) and daughters (G3) born in Sweden by grandmothers’ (G1) region/country of origin

	Mothers (G2)	Daughters (G3)	
	Model 1	Model 1	Model 2
	OR (95% CI)	OR (95% CI)	OR (95% CI)
Sweden (ref)	1	1	1
Non-Western	0.90	1.38**	1.25
	(0.74 to 1.10)	(1.12 to 1.69)	(0.98 to 1.60)
Eastern European	1.02	1.07	0.96
	(0.85 to 1.10)	(0.86 to 1.32)	(0.74 to 1.25)
Western	1.06	1.08	0.98
	(0.85 to 1.32)	(0.85 to 1.38)	(0.73 to 1.33)
Rest of Nordic	1.01	0.96	0.86**
	(0.91 to 1.12)	(0.86 to 1.07)	(0.75 to 0.99)
Constant	1.01	0.80***	0.00*
	(0.97 to 1.05)	(0.77 to 0.82)	(0.0 to 0.84)
Observations	246 642	314 415	314 415

Model 1 includes gestational age (G2 and G3, respectively).

Model 2 adjusted for G3 gestational age, G3 birth year, G3 birth order, G2 birthweight, G2 gestational age, G2 BMI, G2 height, G2 age at birth, G2 smoking during pregnancy, G2 income and G2 education. Model 2 is equivalent to model 4 in linear models.

Standard errors are clustered by mother’s ID for G3 model.

*P<0.05, **p<0.01, ***p<0.001.

Figure 3 (estimates presented in online supplemental table 4) presents the results from the random effects linear models for G3 birthweight differences by G1 origin relative to the corresponding native Swedish population with different levels of adjustments. Although the differences are reduced relative to those observed in Panel A, the above-mentioned patterns were consistent. The only set of controls that substantially reduce the inequalities in G3 are included in model 3 (ie, G2 birthweight, gestational age, height and BMI). This suggests that body compositional differences between groups partially explained differences in birthweight across groups; however, substantial inequality remains even after adjusting for these factors. The non-western G3 group maintained a −85 g difference compared with the G3 descendants of Swedes and continued to show increased odds of low birthweight at 1.25 (95% CI 0.98 to 1.60). Small changes were observed when socioeconomic factors and/or behavioural characteristics (ie, smoking and maternal age) were taken into account for all groups except for the descendants of non-western immigrants (model 4).

Sensitivity analysis (online supplemental table 5) indicated that there is variation across regional groups within our analytical categories in G2 and G3 mean birthweight, respectively, relative to the reference group. However, our categorisation did not mask important subgroup variation in the *birthweight change over generation*. Within the non-western group, Lebanon showed the greatest change in birthweight differences across generations relative to the Swedish population (−153 g difference between G2 and G3). However, an important exception was found among the descendants of Iranian women, whose birthweight difference converged between mothers and daughters to the levels of the native population (by 132 g). Despite the ethnic and geographical diversity of the non-Western group, all but one group display increasing disparities in birthweight over generation relative to the native population.

## Discussion

### Summary of the results

Our study shows growing inequalities across generations in birthweight and low birthweight among individuals born in Sweden with immigrant ancestry. Relative to native Swedes, the descendants of non-Western immigrants show lower birthweight in G2 and even larger differences in G3; whereas, the descendants of Eastern European, Western and rest of Nordic immigrants display no change or reduced inequalities in birthweight over a generation. Growing inequalities in birthweight are due to the non-western group experiencing no improvement in mean birthweight. Moreover, there has been no change in the SD of birthweight suggesting that this uniform divergence is specific to the descendants of non-western immigrants relative to Swedes.

Given that all births occurred in Sweden (G2 and G3), any medical improvements increasing the viability of preterm births or small babies should be systematically shared in an equitable society. Our sensitivity analyses restricted to term births confirm the main results.

### Implication of the findings

Several studies have examined birthweight changes between first-generation and second-generation immigrants from the same country of origin.^10 18^ A previous systematic review and meta-analysis found negligible birthweight differences over generations, however, a majority of studies included did not use intergenerational linkages. More recently, a study using intergenerational linkages in the USA^18^ found that children of foreign-born black women had higher birthweights and lower prevalence of LBW as compared with US-born black women; however, within one generation the perinatal health advantages of foreign-born black women deteriorated and converged with those of their US-born counterparts. Since, similar patterns were not observed among the descendants of other immigrant groups, the authors suggest that exposure to discrimination and socioeconomic inequality is associated with adverse health outcomes for black women.

Our study builds on the aforementioned research by comparing intergenerational changes in birthweight among immigrant groups to the native population in order to understand how inequality in birthweight develops over generation. In line with those studies, we show increasing birthweight between G2 and G3 among all immigrant groups and the native population except among the descendants of non-western immigrants, thus leading to increasing inequality. This finding is puzzling considering that Sweden has been lauded for its humanitarian approach to migration and for being one of the most egalitarian countries in the world with universal access to healthcare and education. Adjustments for socioeconomic conditions did not attenuate the differences between G3 descendants of non-western immigrants and natives, whereas they did—although to a small extent—for other immigrant groups; however, other social factors might be at play, including discrimination and racism,^19^ both of which are salient conditions non-western groups face in Sweden and have been shown to negatively impact health.^20–24^

Increasing inequality in birthweight can possibly be explained by a combination of premigration and postmigration factors influencing the health of immigrants and their descendants. Trauma associated with forced migration (eg, post-traumatic stress disorder) has been shown to have deleterious effects on the health of immigrants, including in low birthweight, which may have intergenerational impacts through epigenetic transmission.^25^ Moreover, G2 in-utero exposures can impact G3 health through epigenetic marks in the germ cells. This transgenerational biological effect might not manifest in G2 nor be easily mitigated by postnatal factors. The non-western and eastern European groups are both comprised of the descendants of ethnically diverse immigrant populations predominantly from countries in conflict. At the same time, however, marked birthweight divergence is *only* observed among the descendants of non-western immigrants, suggesting that postmigration factors influencing perinatal health differ between these two groups and minimise the possibility that epigenetic transmission is the key mechanism. This health pattern is revealed, across generations, to embody racial health inequalities^26^ as we observed an increased divide between white and non-white groups in Sweden.

Our results connect to the broad literature on racial differences in birthweight conducted in countries such as the USA,^18^ Brazil^27^ and the UK.^28–30^ By examining two generations of the descendants of immigrants in Sweden, we show the social roots of racial disparities in birthweight. Moreover, if birthweight is linked to health^31–35^ and cognitive development^36^ throughout the life span and socioeconomic status^37^ in adulthood, as suggested by the life-course research, birthweight could be regarded as an important mechanism driving health inequalities between ethnic/racial minorities and majority populations across the western world.

### Strengths and limitations

To the best of our knowledge, our’s is the first study to show intergenerational patterns of birthweight based on migrant ancestry using total population data and complete multigenerational linkages. This is a clear contribution since most prior studies have been only able to compared birthweight outcomes between first and second-generation migrants using cross-sectional data and, the very few studies with intergenerational linkages,^10^ were restricted to a limited number of origin groups in the USA and the UK that were either non-representative of the immigrant population, underpowered or flawed by selection bias in their sample construction. Likewise, this is the first study to examine birthweight differences between third-generation migrants and natives while adjusting for their maternal perinatal history (G2 birthweight and gestational age).

However, there are some limitations worth noting. We lack coverage on the father’s birthweight, which may be important if paternal birthweight systematically differs by matrilineal region of origin or due to intermarriage in the G2. With respect to the latter, our results would be underestimated. Prior evidence has shown, however, that father–child birthweight correlations are substantially smaller than mother–child correlations.^38 39^ In addition, we are only able to create matrilineal linkages for G2 mothers who were 39 years of age or younger and born in Sweden due to the coverage of the MBR. Although this is a limitation, since it does not cover all childbearing years of the cohorts, our data cover the total population and include all third-generation descendants of immigrants born between 1989 and 2012, whose mothers are born between 1973 and 1996.

Although the lack of G1 birthweight can count as a limitation, it is only problematic in the comparison of the birthweight of G2 descendants of immigrants relative to Swedes. We believe that this is a limitation because estimated differences in birthweight between G3 descendants of immigrants and Swedes attenuate substantially after adjusting for maternal compositional characteristics. Nonetheless, this limitation does not explain the growing inequalities between G2 and G3 descendants of non-Western immigrants and native Swedes.

The lack of information on race and ethnicity is a limitation, particularly for the interpretation that birthweight differences over generations indicate a ‘white’/’non-white’ divide. Despite this, we were able to show a pattern of racial inequalities that develop in the host country among the descendants of immigrants from non-western countries. Considering the non-western group, which is comprised of ethnically and geographically diverse immigrant groups, our findings provide strong evidence for the social roots of racial health inequalities. It can be argued that ethnic differences in birthweight exist as a result of differences in intrauterine growth trajectories.^40^ However, our study does not focus on specific ethnic groups but broad categories based on country of origin. The large ethnic diversity of the non-western migrants in Sweden indicates that differences, if any, are likely not the result of ethnic-group-specific growth trajectories. Moreover, birthweight differences across ethnic groups might explain initial disparities (ie, G2 birthweight between immigrants and natives) yet they are unlikely to explain an increasing pattern of inequalities over generations.

### Public health implication

Our study adopts a population health perspective,^41^ considering not only adverse outcomes (ie, low birthweight) but also the distribution of health outcomes (ie, a continuous measure of birthweight). Although it can be argued that this is of less interest from a clinical perspective, it is worth noting ‘distributional’ differences in birthweight found among the descendants of non-western migrants also reflected higher odds of low birthweight. Furthermore, among the descendants of non-western immigrants, we observe average differences of around 100 g between generations and/or in relation to the native group (eg, the 153 g increase in the birthweight disparity across generations of Lebanese descendants and a 132 g convergence relative to natives among Iranian descendants), which is comparable to the effects of more commonly described risk factors, such as smoking during pregnancy.^42^

In conclusion, our study shows that health inequalities are increasing between the descendants of immigrants and the native population following a white–non-white divide. Given that birthweight is a measure of the intergenerational transmission of health, our findings suggest that without proper intervention inequalities may continue to widen in subsequent generations.

## Data Availability

Data may be obtained from a third party and are not publicly available. Aggregated data can be made available by the authors, conditional on ethical vetting. The authors access the individual-level data through Statistics Sweden’s micro-online access system MONA.
