# Supplementary material for: Immigrant ancestry and birthweight across two generations born in Sweden: an intergenerational cohort study
Source: BMJ Glob Health. 2022 Apr 10;7(4):e007341. doi: 10.1136/bmjgh-2021-007341 (PMC9058695; doi:10.1136/bmjgh-2021-007341)
Supplement: Supplementary data [file bmjgh-2021-007341supp001.pdf]

Supplementary file

Table 1. Birthweight Differences for mothers (G2) and daughters (G3) by grandmothers' (G1) regions of origin relative to native Swedes G1 (estimates corresponding to figure 3. Panel A). From Linear Regression Models.

	**G2**	**G3**
	**Beta [95%CI]**	**Beta [95%CI]**
Sweden (ref)	ref	ref
Non-Western	-80***	-147***
	[-96,-64]	[-163,-132]
Eastern European	-56***	-70***
	[-73,-39]	[-86,-55]
Western	-56***	-63***
	[-75,-37]	[-80,-45]
Rest of Nordics	-11**	8
	[-20,-1]	[-1,17]
Constant	3466***	3549***
	[3464,3468]	[3548,3552]
Adjusted R^2	0.139	0.194
Observations	246 642	314 415

Models adjusted for gestational age (G2 and G3, respectively) (model 1)
Standard errors are clustered by mother's ID for G3 models.
95%CI= 95% Confidence Intervals; * $p < 0.05$, ** $p < 0.01$, *** $p < 0.001$

Table 2. Sensitivity analyses. Birthweight Differences for daughters (G3) by grandmothers' (G1) regions of origin relative to native Swedes G1 in a subsample of term-births From Linear Regression Models.

	**G2**	**G3**
	**Beta [95%CI]**	**Beta [95%CI]**
Sweden (ref)	ref	ref
Non-Western	-89***	-152***
	[-106,-73]	[-167,-136]
Eastern European	-57***	-75***
	[-74,-40]	[-91,-59]
Western	-60***	-66***
	[-79,-41]	[-84,-48]
Rest of Nordics	-13**	8
	[-22,-4]	[-1,17]
Constant	3466***	3549***
	[3464,3468]	[3548,3552]
Adjusted R^2	0.001	0.002
Observations	229 275	297 572

Models without controls.
Standard errors are clustered by mother's ID in both models.
95%CI= 95% Confidence Intervals; * $p < 0.05$, ** $p < 0.01$, *** $p < 0.001$

Table 3. Low birthweight at term (<2,500 grams and 37-42 gestational weeks) differences among mothers (G2) and daughters (G3) by immigrants' mother's (G1) region of origin.

	G2		G3		
	%	Model 1	%	Model 1	Model 2
		OR [95%CI]		OR [95%CI]	OR [95%CI]
Sweden (ref)	1.98	1	1.01	1	1
Non-Western	1.99	1.00	1.47	1.46	1.17
		[0.78,1.28]		[1.12,1.90]	[0.90,1.53]
Eastern European	2.04	1.03	1.21	1.19	1.02
		[0.80,1.33]		[0.89,1.59]	[0.76,1.37]
Western	2.00	1.04	1.20	1.19	1.04
		[0.76,1.34]		[0.86,1.64]	[0.75,1.43]
Rest of Nordics	2.08	1.05	1.00	0.94	0.84
		[0.92,1.20]		[0.79,1.11]	[0.70,0.99]
Observations		229 275		297 572	297 572

Model 1 without controls.
Model 2 adjusted for G3 birth year, G3 birth order, G2 birthweight, G2 gestational age, G2 BMI, G2 height, G2 age at birth, G2 smoking during pregnancy, G2 income and G2 education. Model 2 is equivalent to model 4 in linear models
Standard errors are clustered by mother's ID for G3 model.
OR=Odds Ratios; 95%CI= 95% Confidence Intervals;
* $p < 0.05$, ** $p < 0.01$, *** $p < 0.001$

Table 4. Birthweight differences for daughters (G3) by grandmothers' (G1) regions of origin relative to native Swedes G1 with different model specifications (estimates corresponding to figure 3. Panel B). From Linear Regression Models.

	Model 2	Model 3	Model 4
	Beta [95%CI]	Beta [95%CI]	Beta [95%CI]
Sweden (ref)	ref	ref	ref
Non-Western	-143***	-86***	-85***
	[-158,-128]	[-100,-71]	[-100,-71]
Eastern European	-67***	-46***	-38***
	[-82,-51]	[-61,-31]	[-52,-23]
Western	-58***	-34***	-30***
	[-75,-40]	[-51,-18]	[-47,-14]
Rest of Nordics	6	12**	20
	[-2,14]	[4,-20]	[13,28]
Constant	4651***	5789***	6645***
	[3842,5459]	[5016,6562]	[5752,7537]
Adjusted R^2 (overall)	0.210	0.28	0.29
Observations	314 415	314 415	314 415

Model 2: adjusted for G3 gestational age, G3 birth year and G3 birth order
Model 3: adjusted for G3 gestational age, G3 birth year, G3 birth order, G2 birthweight, G2 gestational age, G2 BMI and G2 height.
Model 4: adjusted for G3 gestational age, G3 birth year, G3 birth order, G2 birthweight, G2 gestational age, G2 BMI, G2 height, G2 age at birth, G2 smoking during pregnancy, G2 income and G2 education.
95%CI= 95% Confidence Intervals; * $p < 0.05$, ** $p < 0.01$, *** $p < 0.001$

Table 5. Mean birthweight differences among mothers (G2) and daughters (G3) by immigrant's mothers' (G1) country or region of birth

	G2	G3		
	Model 1	Model 1	Model 3	Model 4
	Beta [95%CI]	Beta [95%CI]	Beta [95%CI]	Beta [95%CI]
Sweden (ref)	0	0	0	0
**Non-Western**				
Rest of Africa	-84**	-108***	-64*	-66*
	[-144,-24]	[-157,-60]	[-115,-13]	[-117,-15]
East Africa	-22	-90	-34	-33
	[-136,92]	[-232,52]	[-163,94]	[-161,95]
Iraq	-77	-63	26	30
	[-199,46]	[-220,93]	[-90,144]	[-103,129]
Iran	-164**	-32	70	58
	[-274,-53]	[-171,108]	[-53,193]	[-65,180]
Lebanon	-61*	-214***	-146***	-153***
	[-120,-2]	[-265,-162]	[-198,-94]	[-205,-101]
Turkey	-96***	-166***	-110***	-103***
	[-123,-70]	[-190,-142]	[-133,-86]	[-126,-8]
Asia	-100***	-176***	-101***	-103***
	[-132,-69]	[-208,-144]	[-131,-71]	[-133,-73]
Chile	-21	-89***	-31	-36
	[-71,28]	[-135,-43]	[-75,30]	[-81,6]
South America (except Chile)	-24	-51	-8	-5
	[-86,37]	[-120,18]	[-63,48]	[-60,50]
**Eastern Europe**				
Non-EU27 Europe	-46	-51*	-28	-24
	[-98,6]	[-95,-6]	[-74,18]	[-70,21]
Former Yugoslavia	-55***	-89***	-66***	-55***
	[-78,-33]	[-109,-68]	[-86,-46]	[-75,-35]
Poland and former Soviet Union	-62***	-45**	-18	-12
	[-91,-33]	[-72,-18]	[-44,8]	[-38,14]
**Western**				
EU27/North America and Oceania	-56***	-63***	-34***	-30***
	[-75,-37]	[-80,-45]	[-51,-18]	[-46,-14]
**Rest of Nordics**				
Finland	-8	8	14***	22***
	[-18,2]	[-2,18]	[6,23]	[14,31]
Rest of Nordics	-22*	8	3	13
	[-42,-1]	[-11,27]	[-14,20]	[-4,30]
Constant	3466***	3550***	5828***	6666***
	[3464,3468]	[3548,3552]	[5055,6602]	[5772,7559]
Observations	246 642	314 415	314 415	314 415

Model 1 adjusted for gestational age (G2 and G3, respectively)
Model 3 adjusted for G3 gestational age, G3 birth year, G3 birth order, G2 birthweight, G2 gestational age, G2 BMI and G2 height.
Model 4 adjusted for G3 gestational age, G3 birth year, G3 birth order, G2 birthweight, G2 gestational age, G2 BMI, G2 height, G2 age at birth, G2 smoking during pregnancy, G2 income and G2 education.
Standard errors are clustered by mother's ID for model 1. Model 2 and 3 are estimated from Random effect linear models
95%CI= 95% Confidence Intervals; * $p < 0.05$, ** $p < 0.01$, *** $p < 0.001$
